# Supplementary material for: Computational assessment of the feasibility of protonation-based protein sequencing
Source: PLoS One. 2020 Sep 11;15(9):e0238625. doi: 10.1371/journal.pone.0238625 (PMC7485799; doi:10.1371/journal.pone.0238625)
Supplement: Attachment — Submitted filename: Comments-to-Authors_PONE-D-19-32444_Jan-2020.docx [file pone.0238625.s001.docx]

**Comments to the Authors for the manuscript: PONE-D-19-32444**

**1.** In Page 3/16, in the section ‘Protonation-based protein sequencing’, the N-terminal amine is wrongly depicted as (-CH_2_). The N-terminal amine must be (-NH_2_). And therefore, in the reaction shown in the following line: the N-terminal amine: -CH3+ must be corrected to –NH_3_+ and –CH_2_ should be corrected to –NH_2_.

**2.** In pages 3/16 (last line) and 4/16 (first two lines): the authors mention that they identify amino acids through their carboxyl group pKa values. This means that upon ‘Deprotonation’, the carboxyl group would possess Negative charge and on ‘Reprotonation’, the carboxyl group would NOT be charged, viz., Neutral. Further, the authors have defined that ‘Deprotonation’ leads to “OFF” position and ‘Reprotonation’ leads to “ON” position (Figure 3). So, does this mean that formation of ‘NEGATIVELY CHARGED Carboxyl group’ due to Deprotonation, corresponds to OFF state, while formation of ‘Neutral or Uncharged carboxyl group’ due to Reprotonation, corresponds to ON state? Can authors clarify this?

**3.** In Page 5/16, in the ‘Results and Discussion’ section, the authors define the ON and OFF states: “The signal consists of a high voltage, when a proton is bound (ON-state) and a low voltage otherwise (OFF-state) (see Figure 3).” Since, the authors have opted N-terminus of the peptide for anchoring to the substrate, the Carboxyl group of the peptide is Free. And in the case of Free Carboxyl group, the Proton Bound state (i.e., Reprotonation) is actually UNCHARGED, viz., -COOH. So, it is not clear, as to, how the authors define or consider the UNCHARGED Proton Bound form: -COOH, to be ON-state. In other words, how the UNCHARGED form can contribute for high voltage signal? Likewise, it is not clear, how the Deprotonated form of carboxyl group, -COO^-^, which has a single Negative charge can contribute for low voltage, i.e., OFF-state. In other words, it is not understandable, as to, how the authors correlate the CHARGED Form (-COO^-^) to OFF-state (low voltage) and UNCHARGED Form (-COOH) to ON-state (high voltage).

Moreover, in lower pH range of 2 – 5, the population of UNCHARGED form (-COOH) of Carboxyl group (-COOH) would be more than the CHARGED form (-COO^-^), i.e., the UNCHARGED Form would have a greater lifetime than the CHARGED form in lower pH range of 2 - 5. So, would it mean that in lower pH range of 2 – 5, higher frequency of ON-states or higher voltages would be detected due to UNCHARGED form (-COOH), as per the authors’ current definition of ON and OFF-states? If this is the case, how is it possible for the UNCHARGED form (-COOH) to contribute for higher voltage/ON-state? In contrast, when the population or the lifetime of UNCHARGED form (-COOH) is higher, then it should contribute for lower voltage or OFF state, because of absence of charge on the molecule.

Can the authors explain or rationalize this clearly? Without proper explanation or understanding of ON and OFF states, this study would not have any meaning or further applications. So, Figure 3 may require modification, in that, the definitions of ON/OFF states for Free Carboxyl group can be different from the ON/OFF states for Free Amino group, depending on the pH of the medium, in which the peptide is present.

**4.** In Page 13/16, in the section “Underlying terminal reprotonation-deprotonation signal”, from the equations defined for ‘k_on_’ and ‘k_off_’, it can be understood that

**(i)** Larger the difference between pH and pKa would correspond to Lower ‘k_off_’ values, which implies that the peptide would be in the ON-state for a Larger fraction of time.

**(ii)** Lower the difference between pH and pKa would mean Higher ‘k_off_’ values, which implies that the peptide would be in the OFF-state for a Larger fraction of time.

It is well known that the pKa values of free Carboxyl group of C-terminus of the peptide and the pKa values of sidechain carboxyl groups of Aspartic acid (Asp) and Glutamic acid (Glu) are very close to the acidic pH value. And hence, ALL the carboxyl groups would be in the OFF-state for a Larger time fraction. Moreover, at lower pH values, the population of UNCHARGED form (i.e. Deprotonated form) of the carboxyl groups would also be Higher. So, according to the equation proposed by the authors, it is clear that the representation of Protonation and Deprotonation is not correct, as shown in Figure 3. Therefore, Figure 3 must be modified, so as to have agreement with equation proposed for ‘k_off_’. In other words, the authors have defined ‘k_off_’ and ‘k_on_’equations correctly, but these equations do not accurately represent the Proton-bound form and the Deprotonated form, particularly for the free Carboxyl groups.

Overall, the authors are urged to provide clarification for the proper definition of ON-state and OFF-state, by taking into account the conditions that are shown below:

**(i)** for Free Carboxyl Groups (whose pKa values are closer to acidic pH):

Deprotonation = NEGATIVELY CHARGED Form = -COO^‑^ for pH > ~ 2.5 or 3	and

Reprotonation = UNCHARGED Form = -COOH for pH < ~ 2

Therefore, Deprotonation = ON-state (Higher Voltage)	and

Reprotonation = OFF-state (Lower Voltage)

**(ii)** for Free Amino Groups (whose pKa values are higher than acidic pH):

Deprotonation = UNCHARGED Form = -NH_2_ for pH > 10 (approx.) and

Reprotonation = POSITIVELY CHARGED Form = -N^+^H_3_ in the pH range: 2 – 10.

Therefore, Deprotonation = OFF-state (Lower Voltage)	and

Reprotonation = ON-state (Higher Voltage)

Thus, the representation shown in Figure 3 is correct for Free Amino Groups and not for Free Carboxyl Groups. The authors need to make a note of this and do the necessary corrections.

**5.** Digestion of proteins would give rise to many peptides. So, how is it ensured that only One Molecule of the Peptide binds to a chip? Is the chip designed in a manner that only One Molecule of the Peptide can be accommodated on its surface? It is established that chromatographic methods aid in decreasing the complexity of the mixture of peptides. So, before allowing the digested peptides to bind onto the chip surface, is the mixture of digested peptides subjected to any chromatographic method? In other words, are the peptides introduced to bind onto the chip surface, after any chromatographic method? Or, is the mixture of digested peptides directly introduced onto the chip surface for binding?

**6.** The authors need to mention the source, from where the pKa values have been taken to prepare Table 1.

**7.** What do the authors mean by ‘clusters’? It is better to give a proper definition for ‘clusters’ and then describe about them. How a cluster is different from sequence?

**8.** Why the authors have not compared their simulation results with some experimental data that may be already available?
