# Supplementary material for: Computational assessment of the feasibility of protonation-based protein sequencing
Source: PLoS One. 2020 Sep 11;15(9):e0238625. doi: 10.1371/journal.pone.0238625 (PMC7485799; doi:10.1371/journal.pone.0238625)
Supplement: Attachment — Submitted filename: Comments-to-Authors_PONE-D-19-32444_R1_May-2020.docx [file pone.0238625.s002.docx]

**Comments to the Authors for the manuscript: PONE-D-19-32444R1**

**1.** The authors have now clarified the definition of ‘ON’ and ‘OFF’ with respect to the Carboxyl group, whereby they have ‘reiterated’ that ‘Deprotonated state (Negatively charged form)’ corresponds to ‘OFF-state’ and ‘Protonated state (Neutral form)’ corresponds to ‘ON-state’.

But, a doubt that still prevails is that how a ‘Neutral form’ (Protonated form: COOH) can give rise to a higher voltage than the ‘Deprotonated form (Carboxylate form: COO-)’? In other words, how can a Neutral form give a response to the electronic or electric circuit, since the Neutral form does NOT carry any Charge? However, the ‘Deprotonated (Negatively charged) form’ can be detected by the electronic or electric circuit, since it possesses a Charge, although it could show a signal in the negative axis of the output graph, i.e., negative voltage or negative potential. So, can the authors clarify this? If it would have been an experimental work, then this question could be answered in a more convincing manner. However, this is a computational work and not experimental. Therefore, it becomes even more important that such definitions need to be proposed lucidly and properly.

**2.** In page 14/17, in the section, **“Underlying terminal reprotonation-deprotonation signal”**: the equations for ‘ON-time’ and ‘OFF-time’ must be clarified. Currently, the authors have defined as:

(a) ON-time ~ Exp (**k_off_**), and

(b) OFF-time ~ Exp (**k_on_**)

This definition seems to give a wrong impression. As per this equation, when **k_off_** is higher, then ON-time is higher. Likewise, higher the value of **k_on_**, higher will be OFF-time. The authors need to clarify, as to, how is this possible? In other words, how ON-time can be ‘directly proportional’ to **k_off_** and how is it possible for the OFF-time to be ‘directly proportional’ to **k_on_**?

When **k_off_** is a higher value, then the system (i.e., the molecule) would tend to be in OFF-state (i.e., Deprotonated form), MORE Frequently than in the ON-state. In other words, ‘OFF’ state would be detected or observed MORE frequently than the ‘ON’ state. So, the question is: “During a particular time period of measurement, if the ‘OFF-state’ would be detected at a higher frequency than ON-state, then how ‘ON-time’ can be directly proportional to **k_off_**? When ‘OFF-time’ would be observed at a higher frequency than ON-state, it means that the Total OFF-time (also the Average OFF-time) would be greater than the Total ON-time (also the Average ON-time), during a particular time period of measurement, i.e.,

Total OFF-time **>** Total ON-time, which implies, Average OFF-time **>** Average ON-time

Therefore, it is not clear, as to, on what basis or rationale, the authors have proposed these two definitions? The authors have mentioned, “the associative and dissociative events occur continuously, independently and at a constant average rate of **k_off_** and **k_on_**. But, what is the relationship or connection between this statement, i.e., “association and dissociation events occur continuously, independently, at a constant average rate of **k_off_** and **k_on_**”; and the equations defined for ON-time and OFF-time?

**3.** The method proposed in this work is appropriate for **‘pKa measurement’**, rather than for protein sequencing. Further, the proposed strategy may also be applicable to determine ‘isoelectric point’ of protein. However, in the context of protein sequencing, this proposed method of measuring reprotonation and deprotonation events, can become meaningful and useful, only when this method is combined with proteolysis (by using endopeptidase) or by combining with Edman’s method. Another essential requirement is a proper reference database containing protein sequences. So, without involvement of proteolysis or Edman’s method and in the absence of a reference database, this method can only be useful to measure pKa or isoelectric point of proteins. Therefore, what is the need to mention ‘protein sequencing’ in the title of this manuscript? Instead, the authors may change the title, perhaps by emphasizing ‘pKa measurement’ of single molecule of protein. The authors could also add a phrase in the title by mentioning: ‘application to protein sequencing’. So, this manuscript may be titled as “pKa measurement of protein using (carbon nanotube) FET for protein sequencing”. Or it may be titled as, “Single molecule pKa measurement (by carbon nanotube FET) for protein sequencing”. The authors can decide, as to, whether to mention about FET or not, in the title?

Therefore, accordingly, in the list of keywords, ‘pKa measurment’ or ‘pKa’ can be included. Even ‘Isoelecric point (pI)’ can be mentioned in the list of keywords.
