# Supplementary material for: Computational assessment of the feasibility of protonation-based protein sequencing
Source: PLoS One. 2020 Sep 11;15(9):e0238625. doi: 10.1371/journal.pone.0238625 (PMC7485799; doi:10.1371/journal.pone.0238625)
Supplement: Attachment — Submitted filename: Comments-to-Authors_PONE-D-19-32444_R2_August-2020.docx [file pone.0238625.s003.docx]

**Comments to the Authors for the manuscript: PONE-D-19-32444R2**

**1.** In places, where the Protonated Form of Amine group is shown, the POSITIVE CHARGE (**+**) MUST be positioned NEAR the Nitrogen Atom, i.e., as superscript to Nitrogen atom, on it’s left-hand side, as shown here: - **^+^**NH_3_.

Currently, it is shown as ‘superscript’ of the Hydrogen Atom, which is NOT Correct. So, the authors MUST make this correction, before the manuscript is taken up for publication.

**2.** In page 3/17, in the section ‘Protonation-based protein sequencing’, second paragraph,

line no. 8: ‘mass spectroscopy’ must be corrected to ‘mass spectrometry’. So, it must read as ‘mass spectrometry-based proteomics’.

**3.** In page 6/17, second paragraph, line no. 7: …..the voltage difference between the on-state and off-state., can be mentioned within inverted commas, viz., “the voltage difference between the on-state and off-state.” In doing so, this point might get more emphasis, thereby enabling better understanding for the readers.

**4.** In page 14/17, in the section, **“Underlying terminal reprotonation-deprotonation signal”**: clarification for the equations for ‘ON-time’ and ‘OFF-time’ was requested earlier.

(a) ON-time ~ Exp (**k_off_**), and

(b) OFF-time ~ Exp (**k_on_**)

In response to this query, the authors have now added a sentence and included a mathematical function, **f(x) = ke^-kx^**, which was not mentioned in the earlier two versions of this manuscript. By including this mathematical function, there seems to be a better clarity and understanding in the definitions about ON-time and OFF-time. However, the authors are urged to provide some detailed explanation for the **‘k’** in this function, **f(x) = ke^-kx^**.

(a) What is the **‘k’** that is appearing as **co-efficient** to the exponential function?

&

(b) What is the **‘k’** that is placed **within the exponential function along with ‘x’**?

Are these **two** **‘k’** same or different? If these **two ‘k’** are same, does this **‘k’** refer to **k_off_** and **k_on_** in the respective equations for ON-time and OFF-time? If these **two ‘k’** are different, then the authors must use another symbol for the co-efficient of this exponential function. And it seems that the **‘k’ which is placed within the exponential function along with ‘x’**, corresponds to **k_off_** and **k_on_** for the respective equations of ON-time and OFF-time. Kindly clarify.
